# Stem cell-based therapies for neuroretinal degeneration: current landscape and future perspectives

**DOI:** 10.1093/stcltm/szag046

**Published:** 2026-07-27

**Authors:** Sarah Saietz, Filippo Locri, Helena Isla-Magrané, Brandon Mills, Fredrik Lanner, Anders Kvanta

**Affiliations:** Department of Clinical Sciences, Intervention and Technology, Karolinska Institutet, Stockholm, Sweden; Gynecology and Reproductive Medicine, Karolinska University Hospital Huddinge, Stockholm, Sweden; Clinical Neuroscience, Section for Ophthalmology and Vision, Karolinska Institutet, St. Erik Eye Hospital, Stockholm, Sweden; Department of Clinical Sciences, Intervention and Technology, Karolinska Institutet, Stockholm, Sweden; Gynecology and Reproductive Medicine, Karolinska University Hospital Huddinge, Stockholm, Sweden; Clinical Neuroscience, Section for Ophthalmology and Vision, Karolinska Institutet, St. Erik Eye Hospital, Stockholm, Sweden; Department of Clinical Sciences, Intervention and Technology, Karolinska Institutet, Stockholm, Sweden; Gynecology and Reproductive Medicine, Karolinska University Hospital Huddinge, Stockholm, Sweden; Clinical Neuroscience, Section for Ophthalmology and Vision, Karolinska Institutet, St. Erik Eye Hospital, Stockholm, Sweden

**Keywords:** Neuroretinal degeneration, regenerative medicine, cell replacement therapy, photoreceptors, stem cells

## Abstract

Retinal degenerations, comprising a heterogeneous group of disorders culminating in neuroretinal dysfunction, such as inherited retinal dystrophies (IRDs) and age-related retinal degeneration, are among the leading causes of irreversible vision loss worldwide. Restoring visual function through cell replacement therapy represents an attractive, yet challenging, strategy. Candidate retinal cell sources include retinal progenitor cells (RPCs), Müller glia, human neuroretinal stem-like cells (hNRSCs), and pluripotent stem cells (PSCs). In this review, we discuss their translational advantages and limitations, which in turn influence clinical implementation. We analyze critical manufacturing challenges, including GMP scalability, quality assurance, and ethical considerations in donor material sourcing. Experimental criteria for assessing cellular products’ integration into host circuitry and their capacity to restore visual function in preclinical retinal degeneration models must account for donor maturity, host retina architecture, and immune modulation, which determine survival, synapse connectivity, and delivery requirements. Ongoing clinical trials highlight the diversity of cell replacement strategies—from fetal-derived and allogenic progenitor suspensions to laminated PSC-derived retinal sheets—with each approach presenting distinct implications for survival, true integration, and logistical complexity.

## Graphical Abstract

**Graphical Abstract:**
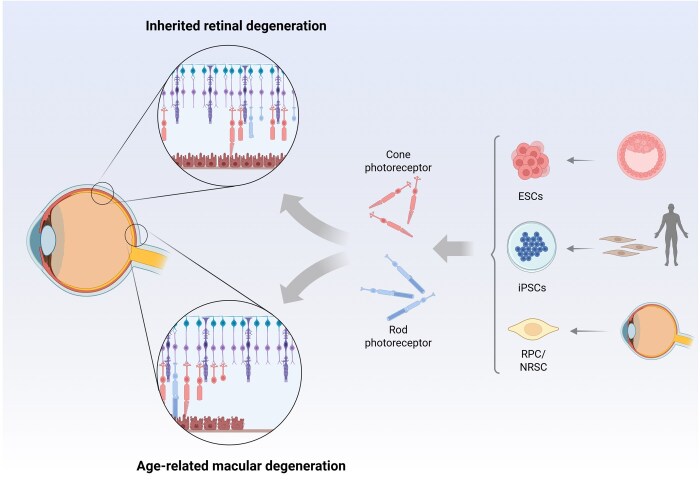

Significance Statement Cell-based regenerative therapies offer a promising strategy to restore retinal structure and function in neuroretinal degenerative diseases. This review surveys diverse retinal cell sources, evaluates the criteria for their integration and functional efficacy in preclinical models, and critically assesses their advantages, limitations, and translational potential to guide clinical implementation.

## Introduction

The progressive degeneration of light-sensing photoreceptors (PhR) is a hallmark of various retinal diseases, most notably blinding conditions such as inherited retinal dystrophies (IRDs) and age-related macular degeneration (AMD). The gradual decline in PhR function leads to visual field loss, progressing from the periphery in most IRDs or from the center in AMD, ultimately resulting in clinical blindness. As the mammalian retina possesses limited endogenous regenerative capacity, cell therapy offers a strategy to replace damaged cells. Human pluripotent stem cell (hPSC)-derived retinal pigment epithelial (RPE) cells, supporting the outer neuroretina, are being evaluated in early-stage clinical trials for conditions such as AMD.^1^^,^^2^ In this review, we focus on strategies for the regeneration of the neuroretina, in particular, lost PhRs.

**Figure 1.**
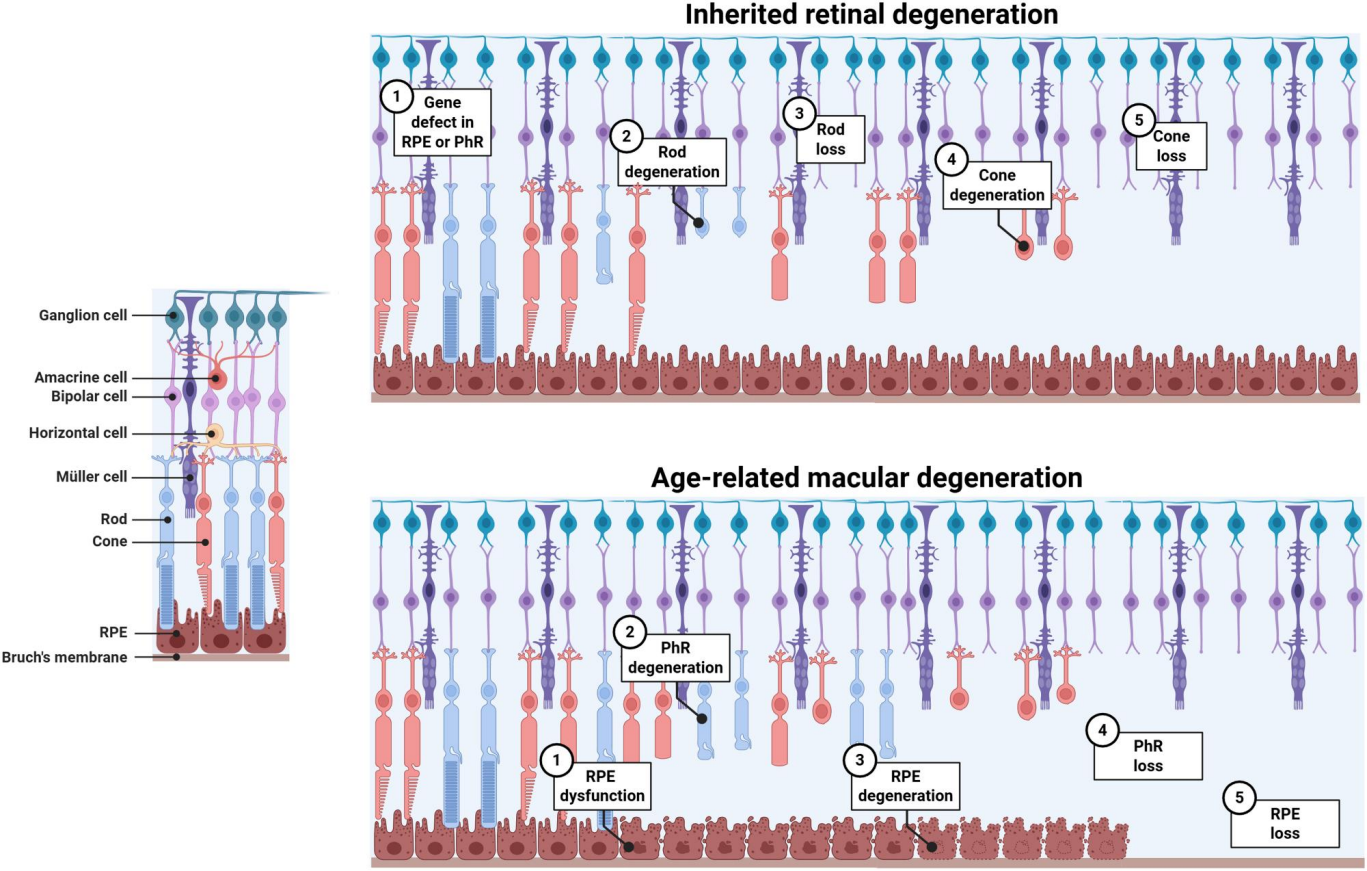
Hallmark pathologic events of the most prevalent retinal degenerative diseases, inherited retinal degeneration (IRD) and age-related macular degeneration (AMD). Abbreviations: PhR: photoreceptor, RPE: retinal pigment epithelium. Created in BioRender. Kvanta, A. (2026) https://BioRender.com/7yy26ct.

Retinal degenerations progressively disrupt neuroretinal function and represent a major cause of permanent vision loss globally. IRDs comprise a heterogeneous group of genetic disorders that affect the retina, commonly manifesting as progressive vision loss due to rod and subsequent cone PhR degeneration (Figure 1).^3^ RP, the most prevalent IRD, involves mutations in over 90 genes and exhibits diverse inheritance patterns and phenotypic variability.^4^ To date, vortigene neparvovec (Luxturna) is the only approved gene therapy for RP.^5^ Despite its efficacy in RPE65-associated RP, its high cost, narrow therapeutic window, and gene-specificity highlight the need for gene-agnostic approaches, such as PhR replacement. The dry form of AMD involves progressive RPE dysfunction leading to initial cone PhR degeneration^6^ and subsequent RPE loss (Figure 1).^7^ Thus, neuroretinal cell replacement therapy, selectively aiming to restore lost PhRs, could, in theory, be sufficient to preserve or improve vision in late-stage dry AMD.

Regenerative medicine seeks to restore or replace cells, tissues, or organs compromised by ageing, disease, or injury. In retinal degenerations, cell-based therapies either provide trophic support to endogenous cells or replace lost cells.^8^ Selecting an appropriate cell source is, therefore, critical and must match the underlying pathology, disease stage, and intended mechanism of action. Early-stage disease may respond to gene therapy or neuroprotective and anti-inflammatory support, whereas advanced disease with extensive cell loss requires direct cellular replacement.

## Cell sources

### Endogenous cells

#### Retinal progenitor cells

**Figure 2.**
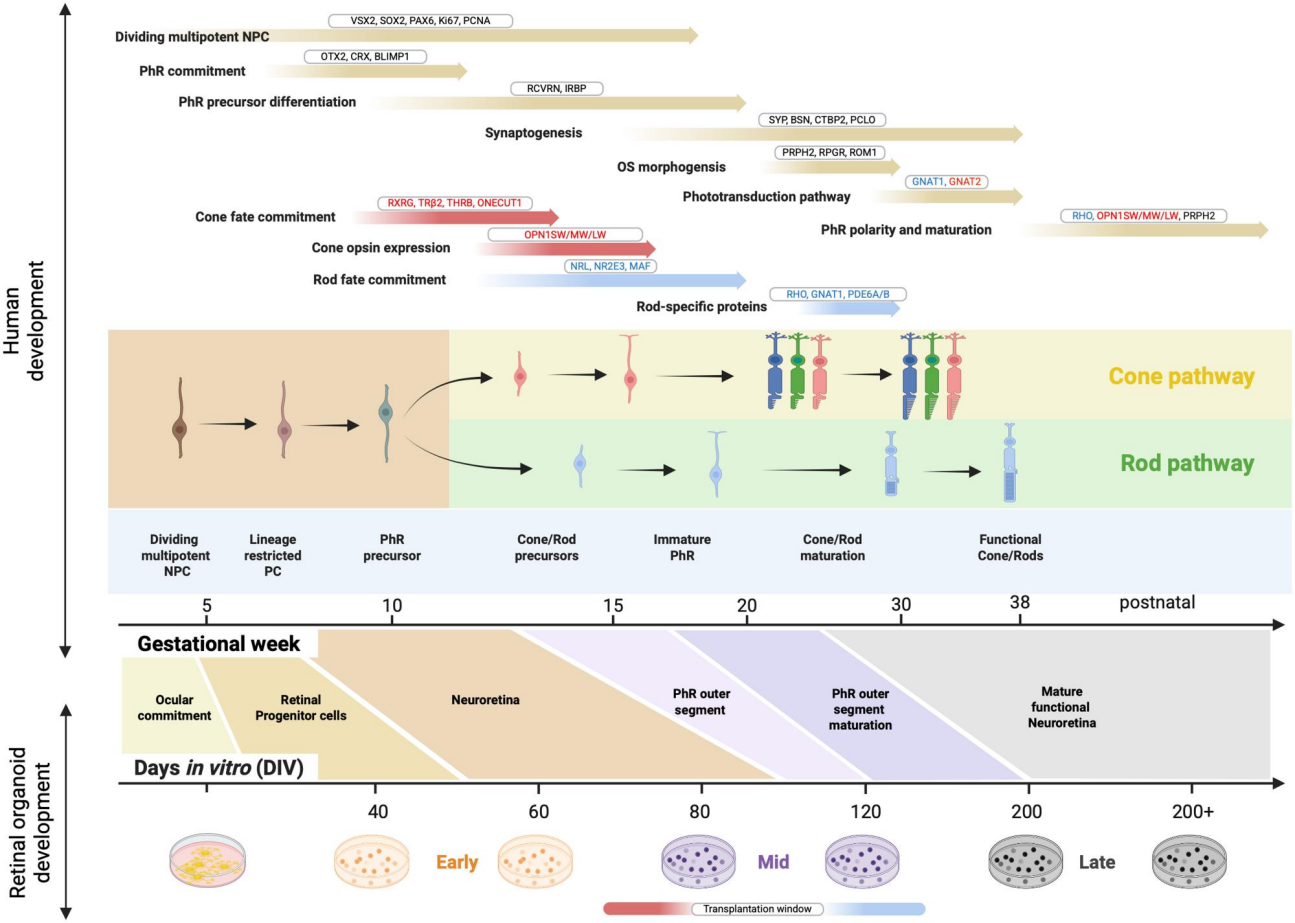
Photoreceptor development *in vivo* and *in vitro*. Human cone (red) and rod (blue) embryonic development stages is shown with the stages most relevant for transplantation highlighted (bottom). Abbreviations: NPC: neuroprogenitor cells, OS: outer segment, PhR: photoreceptor, PC: precursor. Created in BioRender. Kvanta, A. (2026) https://BioRender.com/krkmnh5.

Retinal progenitor cells (RPCs) are multipotent cells capable of orchestrating retinogenesis, giving rise to all major retinal cell types, including PhRs.^9^ While RPCs are lineage-restricted, and they retain notable developmental plasticity and high proliferative potential, making them attractive for cell replacement strategies. RPCs emerge early in development and can be isolated from the human fetal retina between 16 and 20 weeks of gestation (Figure 2). However, the limited availability of donor tissue and the logistical constraints of primary tissue acquisition represent significant translational hurdles.^10^ To this end, human PSC-derived retinal organoids (ROs) have emerged as an alternative, scalable source of RPC-like cells.^11–13^

Preclinical studies have shown that isolated RPCs can survive, migrate into the outer nuclear layer (ONL), and differentiate toward PhRs, in some cases contributing to partial restoration of visual responses.^9^ Compared to mature PhRs, RPCs display a higher integration capacity; however, the disease stage is critical: RPC transplantation is more effective when residual retinal architecture is preserved, likely due to their developmental immaturity and responsiveness to host-derived guidance cues.^14–16^

#### Human neural retinal stem-like cells

The ciliary margin was first identified as a retinal stem cell niche around the turn of the millennium, when self-renewing, multipotent retinal stem cells were isolated from the adult mammalian retina, including humans.^17^^,^^18^ This discovery of postnatal stem cells challenged the long-held view that the mammalian retina lacks regenerative capacity, paving the way for the identification and characterization of additional stem-like populations within this niche.

Recently, a putatively distinct population termed human neural retinal stem-like cells (ss) was identified in the ciliary margin zone of both human fetal peripheral retina and hROs, localized at the interface between the RPEs and neuroretina.^19^^,^^20^ hNRSCs and classical RPCs can both be derived from the developing retina and human ROs, however, available data suggest that hNRSCs are molecularly and functionally distinct. hNRSCs are distinguished from RPCs by their substantial capacity for self-renewal. While RPCs are transiently amplifying progenitors with restricted proliferation, hNRSCs can undergo numerous cell divisions and are continually replenished during retinal growth. hNRSCs have been described to be able to give rise to RPCs, which in turn differentiate into PhR precursors and mature PhR. Molecularly, hNRSCs are identified by the transcription factor MECOM, while functionally, RO-derived hNRSCs transplanted into rd10 mice were shown to efficiently integrate into the host neural retina, differentiate into mature retinal cell types, establish synaptic connections, and promote PhR survival. Additionally, they have been associated with a reduction in host retinal inflammation markers, suggesting a potential role in modulating the inflammatory response. Together, these features—self-renewal, multipotency, and regenerative potential—position hNRSCs as a promising cell source for next-generation therapies for retinal degenerative diseases.

#### Müller glia

Müller glia span the entire retinal thickness and provide structural, metabolic, and homeostatic support.^21^^,^^22^ A subset of adult human Müller glia expresses neural progenitor markers (nestin, Sox2, Pax6, Chx10) and exhibits the capacity to self-renew, indicating stem cell-like properties.^21^ In contrast to exogenous cell sources, Müller glia offer a promising target for direct reprogramming, for example, through Ascl1, to generate amacrine, bipolar, and PhR-like cells in injury settings.^23^ This endogenous reprogramming strategy may offer advantages over classical cell-based replacement therapies by potentially circumventing challenges related to cell manufacturing, delivery, and post-transplant survival.

### Pluripotent stem cells

Human embryonic stem cells (hESCs), derived from the inner cell mass of donated human blastocysts, and induced pluripotent stem cells (iPSCs), reprogramming adult somatic cells,^24^^,^^25^ can be differentiated into retinal lineages through stepwise protocols that recapitulate embryonic eye development (Figure 2). The generation of hPSC-derived retinal cell types has evolved from early low-efficiency 2D differentiation approaches^26^ to complex 3D systems. A key advancement was the development of self-organized optic cup-like structures by the Sasai laboratory.^11^^,^^27^ Subsequent protocols combined 2D and 3D culture phases, optimizing differentiation outcomes.^12^^,^^13^ Collectively, these advances now enable the generation of laminated, PhR-rich retinal tissue with higher reproducibility,^28^ capable of forming synaptic connections upon transplantation.^12^^,^^16^^,^^29^^,^^30^ Patient-derived iPSC models have also been applied to study IRDs, enabling personalized disease modeling and drug screening.^24^ Moreover, genome editing combined with iPSC-derived retinal tissue production offers a potential route for autologous, mutation-corrected cell replacement therapies.^29^ Preclinical studies demonstrate that PSC-derived PhRs can partially restore host retinal physiology by integrating into the recipient retinal architecture, forming synaptic connections, and improving visual responses such as light sensitivity and temporal resolution.^31^^,^^32^

### Translational comparison of cell sources

The translational potential of candidate cell sources reflects trade-offs among scalability, integration, immunogenicity, ethics, and clinical readiness. RPCs are the most advanced (Phase I/II) with good safety profiles, but reliance on fetal tissue limits scalability and raises ethical concerns.^10^ hPSC-derived sources (eg, organoid-derived RPCs, hNRSCs, and photoreceptor progenitors) improve scalability and standardization but involve manufacturing complexity^14–16^^,^^19^^,^^20^ and potential immunogenicity,^11^ with variable integration depending on developmental stage and host environment. Müller glia offer an endogenous, biocompatible, and non-immunogenic option without manufacturing demands, but are limited by poor regenerative capacity in humans, reprogramming challenges, and glial scarring, keeping them preclinical.^22^^,^^23^

Overall, hPSC-derived photoreceptors may suit late-stage degeneration, Müller glia early-stage disease, and progenitors intermediate stages with remaining retinal structure. After selecting a cell source, the key challenge is developing a clinically viable and effective cell product.

## Cell replacement challenges

Cell-based advanced therapies for retinal degeneration must overcome a series of hurdles to achieve successful clinical translation. These challenges span both the production of the cell product itself, the *in vitro* challenges, and those encountered following transplantation, referred to as the *in vivo* challenges. In the coming sections, key barriers are discussed, with an emphasis on translational considerations relevant to clinical implementation.

### *In vitro* challenges

Successful clinical translation of retinal cell replacement therapies depends on overcoming a range of *in vitro*-related challenges that directly impact product safety, efficacy, and scalability. These involve the selection of an appropriate cell source, reproducible generation of the desired retinal cell type at scale, elimination of off-target or tumorigenic cells, and compliance with Good Manufacturing Practice (GMP) and regulatory frameworks.

Cell-based therapies may be autologous or allogeneic, each presenting advantages and constraints. While autologous products are theoretically immune-tolerated, they are limited by scalability, cost, and potential disease-related dysfunction. In contrast, allogeneic approaches require more stringent quality control (QC) and immunological risk mitigation but offer greater standardization and accessibility.^33^ Although GMP scalability, standardized QC, and ethical sourcing of donor material have been highlighted as critical determinants of clinical translation, additional regulatory considerations for hPSC-based products have been comprehensively reviewed elsewhere.^34^

Translating a research-grade differentiation protocol into a clinical-grade process remains a major bottleneck that requires GMP-grade raw materials, validated cleanroom facilities, trained personnel, standardized operating procedures, and comprehensive documentation systems. In parallel, critical quality attributes (CQAs), including identity, purity, potency, viability, and genomic stability, to mention a few, must be defined and assessed using validated in-process and release assays.^34–36^ Defining CQAs for hPSC-derived PhR products is particularly challenging due to the lack of standardized functional potency assays that reliably predict *in vivo* integration or synaptic connectivity and remains a substantial translational hurdle for the field.^37^ Advanced transcriptomic approaches, such as RNA sequencing, are increasingly employed to characterize product heterogeneity, support batch-to-batch consistency, and identify predictive biomarkers linked to clinical outcomes.^38^ These measures are of particular importance for allogeneic products due to the substantial level of regulatory scrutiny applied to them.

Scalability represents an additional *in vitro* challenge. Many current differentiation protocols rely on prolonged culture durations, intricate manual handling steps by highly trained personnel, and complex media formulations, raising concerns regarding cost, reproducibility, and feasibility for widespread clinical use.^39–41^ As most hPSC-based differentiation protocols aim to recapitulate human developmental timelines, the extended culture periods to obtain mature PhRs or their precursors increase the cumulative risk of variability or batch failure. This is particularly pronounced for tissue-based products (ROs), which require continuous maintenance, frequent media exchanges, and repeated in-process control assessments. Consequently, many current strategies favor the transplantation of immature retinal tissue (eg, RPCs or PhR precursors), relying on post-transplantation maturation *in vivo* to reduce the logistical and regulatory burden associated with completing PhR differentiation entirely *in vitro*.

To improve scalability and mitigate variability and personnel-associated failure (eg, contamination leading to batch rejection), automation and closed-system manufacturing platforms have emerged as critical enablers of translation. These systems reduce operator-dependent variability and facilitate scale-up or scale-out strategies.^42^^,^^43^ Early implementation of automated systems, such as bioreactor-based culture and robotic handling, is increasingly viewed as essential for commercial viability.

Product heterogeneity and cell purity remain central safety concerns. While hPSC-derived RPE cells can be produced with high purity (>95%-98%) using optimized culture conditions,^44^ hPSC-derived cultures intended for PhR replacement are inherently heterogeneous and require enrichment prior to transplantation. Residual pluripotent cells pose a risk of tumorigenicity, and robust enrichment strategies are therefore essential to eliminate undifferentiated and off-target cells while preserving therapeutic potency. To this end, cell isolation strategies based on molecular markers have been extensively explored. While fluorescent reporter lines enable efficient purification in experimental settings,^30^^,^^45^^,^^46^ they are not clinically translatable due to the genetic modification and subsequent regulatory concerns.^47^ Surface marker-based sorting offers a more clinically compatible alternative, and numerous markers have been identified for retinal cell characterization and enrichment.^48^ However, limited marker specificity and sorting efficiency remain significant obstacles to the generation of homogenous, transplantable bona fide PhRs.^49–51^ Optimized dissociation protocols have improved the recovery and stability of fragile post-mitotic PhRs,^52^ facilitating more efficient downstream sorting and potentially reducing reliance on additional purification steps. Together, these advances illustrate how improvements in upstream tissue processing can complement established marker-based approaches to meet regulatory standards for clinical-grade products.

Collectively, translational considerations increasingly shape *in vitro* product design beyond manufacturing alone. Ethical sourcing of donor material, immunogenicity, and the choice between autologous and allogeneic strategies all influence manufacturing complexity, QC requirements, and regulatory pathways.^33^ Anticipating downstream clinical constraints during process development is therefore essential to ensure that retinal cell products are not only biologically effective but also manufacturable, safe, and accessible.

### *In vivo* challenges

While the presented *in vitro* challenges are critical prerequisites for clinical translation, they do not fully reflect the structural and spatial demands imposed by the degenerating retina *in vivo*. These considerations have driven the development of tissue-based transplantation strategies, such as retinal sheet grafts, designed to better support cell survival, preserve spatial organization, and promote integration post-transplantation.^53^ Although hROs recapitulate key features of retinogenesis and generate functional retinal cells, including PhRs, their spherical architecture poses challenges for subretinal delivery, where a planar, laminated tissue configuration is preferred. To address this, two main strategies have emerged: (i) engineering planar retinal tissue using biomaterial scaffolds,^54^ and (ii) generating sheets by sectioning mature hROs.^55^^,^^56^ Laminated retinal sheet grafts and single-cell suspensions each have distinct strengths and limitations *in vivo*. Retinal sheets preserve tissue architecture and spatial organization, which may support photoreceptor survival and integration, but they are heterogeneous, harder to control in donor maturation, and technically demanding to manufacture and surgically deliver—especially in fragile, degenerating retinas where orientation must be maintained.^47^ In contrast, single-cell suspensions are easier to produce as a defined population and simpler to deliver, but they lack laminar organization and often integrate less effectively.^57^ Overall, this reflects a trade-off between architectural fidelity and practical feasibility: retinal sheets favor structure at the cost of complexity, while single-cell suspensions favor simplicity at the cost of structural organization and potentially reduced integration and survival.

While it is important to consider establishing an appropriate cell product (eg, isolated PhRs), choosing the delivery method (eg, single-cell suspension), it is important to note that the success of the therapy is further shaped by donor cell properties and the host retinal environment. Donor cell competence and developmental stage are key determinants of integration. In mice, immature NRL-expressing post-mitotic rod precursors integrate more efficiently than pre-mitotic progenitors, highlighting a narrow developmental window that permits migration into the host ONL and subsequent maturation.^58^ hPSC-derived PhR precursors can recapitulate this process and elicit light-evoked responses when purified and transplanted in murine models.^37^^,^^59^ Functional rescue further depends on donor-host synaptic integration. Successfully integrated donor rods and cones can form synapses with bipolar cells, restoring retinal and behavioral responses, demonstrating that repair of synaptic circuitry is achievable when donor stage and host architecture are permissive.^14^^,^^46^

Once transplanted in the degenerating retina, donor cells encounter significant metabolic stress. In a canine xenotransplantation study,^60^ transplanted PhRs underwent metabolic reprogramming, likely reflecting a transition from nutrient-rich culture conditions to the comparatively nutrient-poor subretinal space, ultimately leading to cell death. These findings suggest that strategies designed to enhance metabolic resilience, such as the use of hydrogels or scaffolds, may help mitigate metabolic limitations following transplantation.

The host retinal microenvironment further shapes transplantation outcomes. The outer limiting membrane, formed by adherens junctions between PhRs and Müller glia, constitutes a physical barrier to nuclear translocation from the subretinal space; disruption of this complex enhances donor somal entry into the ONL.^61^^,^^62^ In parallel, retinal degeneration triggers progressive neuroretinal remodeling, including dendritic retraction, synaptic rewiring, and glial hypertrophy, further narrowing the temporal window for meaningful donor-host connectivity.^63^

Evaluating *in vivo* outcomes is challenging, as true graft integration must be distinguished from indirect rescue mechanisms. Reporter signals and proteins can transfer from donor cells into host PhRs via material transfer, producing donor-like molecular signatures without full somal integration.^64^^,^^65^ Tunneling nanotubules have been identified as the predominant mechanism underlying this phenomenon, rather than extracellular vesicles or phagocytic uptake. These nanotubules enable contact-dependent cargo exchange through cytoskeletal protrusions, possibly further promoted by subretinal injection-induced retinal detachment and subsequent injury- or inflammation-associated signaling.^66^^,^^67^ The translational relevance of material transfer depends strongly on the transplantation model. In mouse-to-mouse allogeneic transplantation, material transfer is increased in degenerating retinas compared with wild-type hosts.^68^ In contrast, minimal interspecies material transfer has been reported in human-to-mouse xenotransplantation experiments.^45^^,^^46^ Irrespective of recipient background or maturity, human PhRs did not transfer cytoplasmic material to mouse host retinal PhRs. Similarly, co-culturing of human and mouse PhRs showed no evidence of interspecies material transfer, although material exchange was observed among disassociated human PhRs.^69^

Consequently, verification of bona fide integration requires a multi-modal analytical approach. Correct localization of the donor nuclei within the host ONL can be demonstrated using nuclear-restricted reporters.^64^ Synaptic connectivity with the host second-order neurons should be confirmed by visualization of donor-derived ribeye protein complex puncta juxtaposed with mGluR6-positive postsynaptic sites of bipolar cells. And finally, graft-derived physiological function can be assessed by reversibly abolishing glutamatergic responses in multi-electrode array recordings, providing functional evidence of donor-host synaptic connectivity.^45^ Compounding these interpretive challenges is the frequent formation of rosettes, reflecting a disorganized retinal architecture that disrupts PhR polarity and outer segment alignment, thereby limiting functional recovery.^70^^,^^71^ Preventing rosette formation remains a critical objective in PhR transplantation strategies. Although introducing additional complexity as mentioned previously, biomaterial scaffolds may offer structural support to improve cellular organization and reduce rosette formation.

Safety and immunological constraints further influence graft survival and the durability of the therapy. Although the subretinal space exhibits partial immune privilege, allogeneic grafts remain susceptible to host rejection in the absence of appropriate immunomodulation.^72^ In clinical studies, HLA-matched allogeneic iPSC-derived RPE cells survived for up to 12 months without systemic immunosuppression, although local steroid therapy was required in one case following evidence of immune rejection.^73^ In a canine model, systemic immunosuppression combined with topical therapy enhanced the survival and synaptic connectivity of hESC-derived PhR precursors,^74^ indicating that immune-mediated graft loss remains a relevant, albeit low-level, risk *in vivo*.

The inherent risk of tumorigenicity in stem cell-based therapies is closely linked to the developmental potency of the transplanted cells. Residual pluripotent cells can drive neoplastic growth, as demonstrated by teratoma formation following intravitreal injection of undifferentiated ESCs in the rd12 mouse model of retinal degeneration, whereas committed neuroretinal progenitors do not induce such pathology.^75^ Encouragingly, multiple studies have demonstrated the preclinical and clinical safety of differentiated hESC-derived RPE cells delivered as suspensions or monolayer patches.^76^^,^^77^ Similarly, transplantation of post-mitotic PhRs has not resulted in tumor or teratoma formation in neither rodent^45^^,^^78^ nor canine models of retinal degeneration.^74^ In contrast, a clinical case involving iPSC-derived RPE revealed copy number alterations that halted transplantation, underscoring the necessity of stringent release testing to ensure terminal differentiation and genomic stability prior to transplantation.^79^ Together, these findings highlight both the apparent safety of post-mitotic retinal cell grafts and the critical importance of robust preclinical validation and release criteria.

Taken together, the success of retinal cell replacement is dependent on the coordinated optimization of donor cell competence, delivery strategy, host environment, metabolic resilience, safety, and immune control. Many of these variables are only partially predictable from preclinical models and interact in ways that are difficult to fully capture experimentally. As a result, early-stage clinical trials represent a critical translational milestone: not only to establish patient safety, but also to test how different product designs and transplantation strategies perform within the human retina. These considerations frame the rationale for the clinical studies discussed in the following section.

## Key translational insights from cell replacement clinical trials

Building on the translational challenges discussed above, early clinical trials of retinal cell replacement have adopted diverse strategies to balance safety with the potential for functional benefit of neuroretinal regeneration in IRDs and advanced dry AMD. Preclinical studies of neuroretinal cells derived from hPSCs and other progenitor populations have demonstrated encouraging integration and functional rescue, supporting translation to inspiring early-phase trials (summarized in Table 1).

**Table 1.** Cell replacement clinical trials for neuroretinal degenerations.

Trial/Sponsor	Disease	Cell source	Product & Maturity	Delivery method/Route	Clinical phase	Summary of endpoints	Preliminary outcomes/Status	References
**ReNeuron**	RP	fRPCs	Fresh, hRPC suspension	Subretinal injection (PPV)	Phase I/IIa (NCT02464436)	**Primary:** safety/tolerability (ocular/systemic AEs). **Secondary:** exploratory visual function (BCVA, retinal sensitivity).	Safe; possible functional improvement; program discontinued	Luo et al., 2014^81^; Dugel 2020^82^
**jCyte/Santen jCell**	RP	fRPCs	Cryopreserved allogeneic RPCs; jCell suspension	Intravitreal injection	Phase IIb complete (NCT03073733); Phase III planned	**Primary:** safety/tolerability (ocular/systemic AEs). **Secondary:** exploratory visual function (BCVA, contrast sensitivity, visual field, low-luminance mobility, patient-reported visual function)	Higher dose improved VA; well-tolerated	Yang et al., 2024^80^; Yang et al., 2025^83^
**CiRA/RIKEN/Kobe Eye Center**	Advanced RP	iPSC-RPCs	Fresh, 3D laminated retinal sheets; organoid matured *in vitro* to day 70-90	Subretinal implantation (PPV)	Phase I (jRCTa050200027)	**Primary:** safety/tolerability (ocular/systemic AEs) and graft survival. **Secondary:** exploratory visual function (BCVA, visual progression vs untreated eye, retinal thickness at transplant site).	Graft survived ≥2 yrs; no tumorigenesis; localized retinal thickening	Hirami et al., 2023^84^
**BlueRock/FUJIFILM OpCT-001**	Primary PhR diseases incl. RP	iPSC-PhR prog.	Fresh, enriched precursors; suspension	Subretinal injection (PPV)	Phase I/IIa (CLARICO, NCT06789445)	**Primary:** safety/tolerability (ocular/systemic AEs). **Secondary:** exploratory visual function (BCVA, visual function measures, functional vision, anatomic engraftment measures).	First patient dosed 2025; FDA Fast Track and Orphan Drug Designation	Ilic D, et al., 2025^85^; ClinicalTrials.gov, 2025^86^

Abbreviations: AEs: adverse events, BCVA: best corrected visual acuity, fRPC: fetal-derived retinal progenitor cells, iPSC: Induced pluripotent stem cell, PhR: photoreceptor, PPV: pars plana vitrectomy, RP: retinitis pigmentosa.

Progenitor-based suspension therapies, such as fetal-derived RPCs (ReNeuron, jCyte), are delivered subretinally or intravitreally and are proposed to act primarily via neurotrophic mechanisms.^80^ These therapies demonstrate favorable safety, scalability, and preliminary functional benefits, emphasizing the clinical viability of minimally invasive suspension approaches, though they do not directly replace host photoreceptors. Laminated iPSC-derived retinal sheet grafts (RIKEN) aim to preserve tissue architecture and enhance structural integration but introduce greater surgical and manufacturing complexity. Intermediate strategies, such as enriched iPSC-derived photoreceptor suspensions (BlueRock/FUJIFILM), attempt to balance biological specificity with practical delivery considerations.

Collectively, these trials highlight the trade-off between clinical practicality and biological complexity: simpler suspension-based therapies are more readily translatable, while structurally complex grafts may be required for durable vision restoration. These insights underscore the importance of aligning cell type, delivery format, and developmental stage with disease context to optimize reproducible integration, functional recovery, and scalable clinical translation. Outcomes from ongoing studies will be critical in defining best practices for future IRD therapies.

## Conclusion and future perspectives

While conventional therapies primarily aim to manage symptoms or slow disease progression, regenerative approaches seek to restore lost function, such as vision in retinal degenerative disorders. Early efforts in the field largely focused on transplanting of hPSC-derived RPE cells for AMD. More recently, however, emphasis has shifted toward neuroretinal restoration, particularly PhR replacement for IRDs. As most current strategies rely on allogeneic cell sources, genetic engineering approaches to generate hypo-immune grafts capable of evading both adaptive and innate immune rejection are actively being developed and are expected to advance further. A related and especially promising direction is the development of gene-edited autologous neuroretinal or PhR grafts for IRDs caused by defined genetic mutations. This rapidly evolving area encompasses a diverse array of transplantation strategies—including neuroretinal cells or progenitors delivered in suspension, cell sheets, and full-thickness retinal grafts—reflecting the complexity of neuroretinal regeneration. Transplanting PhRs as organized sheets, rather than as dissociated cells, may, for example, reduce rosette formation and improve structural integration and functional outcomes. Neuroretinal regeneration is also likely to be explored in AMD, where PhR degeneration typically precedes RPE loss, either as a standalone PhR replacement approach or in combination with RPE transplantation. Achieving proper integration and laminar organization will likely require “sandwich” cell-sheet strategies, potentially incorporating bioengineered scaffolds that function as synthetic Bruch’s membrane. The identification of RPCs, including hNRSCs, as putative bona fide retinal stem cells, positions them as an attractive alternative cell source. At the same time, this highlights the need to rigorously define cell identity, to determine the optimal stage of maturation for transplantation, and to systematically evaluate integration and functional efficacy. Finally, irrespective of the target disease or cell source, defining appropriate QC assays and release criteria, and ensuring batch-to-batch consistency are complex and resource-intensive processes that should not be underestimated for successful clinical translation.

## Data Availability

Data sharing is not applicable to this article as no new data were created or analyzed in this study.

## CRediT

Sarah Saietz (Conceptualization [Equal], Writing—original draft [Equal], Writing—review & editing [Equal]), Filippo Locri (Writing—original draft [Equal], Writing—review & editing [Equal]), Helena Isla-Magrané (Writing—review & editing [Equal]), Brandon Mills (Writing—review & editing [Equal])
